# Striatal and hippocampal contributions to flexible navigation in rats and humans

**DOI:** 10.1177/2398212820979772

**Published:** 2020-12-21

**Authors:** Christoffer J. Gahnstrom, Hugo J. Spiers

**Affiliations:** Institute of Behavioural Neuroscience, Department of Experimental Psychology, Division of Psychology and Language Sciences, University College London, London, UK

**Keywords:** Spatial navigation, dorsal striatum, hippocampus, flexible behaviour, goals, reinforcement learning, wayfinding

## Abstract

The hippocampus has been firmly established as playing a crucial role in flexible navigation. Recent evidence suggests that dorsal striatum may also play an important role in such goal-directed behaviour in both rodents and humans. Across recent studies, activity in the caudate nucleus has been linked to forward planning and adaptation to changes in the environment. In particular, several human neuroimaging studies have found the caudate nucleus tracks information traditionally associated with that by the hippocampus. In this brief review, we examine this evidence and argue the dorsal striatum encodes the transition structure of the environment during flexible, goal-directed behaviour. We highlight that future research should explore the following: (1) Investigate neural responses during spatial navigation via a biophysically plausible framework explained by reinforcement learning models and (2) Observe the interaction between cortical areas and both the dorsal striatum and hippocampus during flexible navigation.

## Flexibility during goal-directed behaviour

Flexible adaptation in response to unexpected changes in the environment is a central challenge in navigation. Tolman et al. (1946) adeptly illustrated this in his seminal work exploring the capacity of rodents to accommodate detours and adopt shortcuts in complex mazes. This work led to the proposal of the cognitive map hypothesis for flexible behaviour, by which the brain constructs an internal representation of the environment to support navigation (Tolman, 1948). Subsequent neuroscientific research led O’Keefe and Nadel (1978) to propose the hippocampus is primarily responsible for supporting this cognitive map. Particularly central to this proposal is the existence of ‘place cells’ in the hippocampus that show spatially localised activity patterns linked to boundaries and landmarks in an environment (O’Keefe and Dostrovsky, 1971). This was followed by the discovery of a variety of other spatial coding cells supporting navigation (see Grieves and Jeffery, 2017 for review). Given the ubiquity of spatial representation in the hippocampus and neighbouring parahippocampal structures, several essential questions arise: (1) How is information used during flexible navigation, as suggested by the hypothesis of the cognitive map? (2) What information does the hippocampus transmit to downstream regions during navigation? (3) What contributions might other regions of the brain’s navigation systems, such as the dorsal striatum, have for flexible navigation?

Rodent studies lesioning dorsal striatum and hippocampus provide strong evidence for dissociable behavioural strategies related to intact function of these regions during spatial navigation (Andersen et al., 2006; White and Donald, 2002). ‘Place learning’ is a flexible process by which an animal learns associations between distal cues and goal locations in the environment, while response learning is an inflexible process whereby an animal learns a series of actions or responses necessary to reach the goal. Place learning can be investigated using the Morris water maze, a task that targets behavioural flexibility and spatial memory (Devan and White, 1999; McDonald and White, 1994; Morris et al., 1982; Pearce et al., 1998; Whishaw et al., 1987). By the original task protocol, a rat is placed at a pseudo-random location within a cylindrical arena filled with opaque water. No local cues other than distal landmarks and boundary distance are provided. Safety is achieved by swimming to a fixed platform located just below the opaque surface, hidden from view. Escape latencies record time to reach the platform during training as well as during probe trials (when the hidden platform is removed). Lesion or inactivation of the hippocampus impacts place learning by increasing escape latencies compared to that of non-lesioned controls (Morris et al., 1982, Moser et al., 1995; Sutherland et al., 1983). However, lesions in dorsal striatum impair simple approach behaviour when the platform is visible, and instead, rats will swim to previously learned platform location (McDonald and White, 1994).

A paradigm called Delayed-Matched-to-Place further extended the Morris water maze by investigating one-shot learning, a hallmark of behavioural flexibility (Steele and Morris, 1999). In this version of the task, the location of the hidden platform changes each day. This results in a substantial drop in escape latency between the first and second trials. The subsequent trials exhibit latency improvement, but to a much smaller extent. This concept of one-shot learning is an impressive quality of cognitive flexibility difficult to capture by biophysically plausible modelling of place cells (Foster et al., 2000). However, reinforcement learning (RL) can capture this behavioural phenomenon by further simulating cells which estimate real world coordinates (Foster et al., 2000; Tessereau et al., 2020). Together, these simulated cells form an allocentric coordinate system receiving input from the place cells. This coordinate system lacks a biological basis, although this may be analogous to information represented by grid cells in the entorhinal cortex (Hafting et al., 2005). Likewise, simulated deep RL agents endowed with grid-like representation can perform flexible spatial navigation tasks such as the Morris water maze (Banino et al., 2018). In addition, bilateral lesions to the fornix impairs performance in an eight-arm radial maze task, in which rats are trained to revisit certain arms consistently baited with food (Packard et al., 1989). Intact hippocampal function is necessary for place learning in a plus-maze task as well (Packard and McGaugh, 1996). Evidence from neuroimaging studies of humans and patients with hippocampal damage further implicates the hippocampus in supporting both place learning and flexible navigation of novel routes and environments (Bohbot et al., 2007; Hartley et al., 2003; Howard et al., 2014; Iaria et al., 2003; Javadi et al., 2019a, 2019b; Javadi et al., 2017; Patai et al., 2019; Spiers et al., 2001a, 2001b; Spiers and Maguire, 2006; Xu et al., 2010).

In addition to place learning, animals also utilise ‘response learning’, that is, learning based on the responses required to reach the goal (Packard and McGaugh, 1996). Such response learning is shown to depend on the functional integrity of the dorsal striatum (Packard et al., 1989; Packard and McGaugh, 1996). Subsequently, human neuroimaging research has provided convergent evidence for the involvement of the dorsal striatum in such response strategy navigation (Hartley et al., 2003; Iaria et al., 2003; Voermans et al., 2004). Response learning is not traditionally considered flexible because it is tied to the specific features of the environment (e.g. always turn right at the crossroad). By contrast, place learning is thought to be flexible since it is possible to use viewpoint-independent information from the environment to accommodate detours and identify shortcuts and because it does not rely on the presence of a single specific cue.

Recent studies have begun to explore how different types of spatial information may be tracked by specific brain regions during navigation. Two important metrics for flexible navigation are vector-to-goal and path-to-goal (Bicanski and Burgess, 2020; Chadwick et al., 2015; Spiers and Barry, 2015). Using in situ learning experience and film simulation of Soho in London (UK), Howard et al. (2014) identified neural correlates of path distance to goal in the right posterior hippocampus. Such correlates of distance to goal have also been observed in dorsal hippocampal recordings in rats (Spiers et al., 2018) and bats (Sarel et al., 2017). During detour events, the human posterior right hippocampus was also found to track the increase in path distance when a forced detour occurred (Howard et al., 2014). Based on this finding and other evidence from rats (e.g. Gupta et al., 2010; Ólafsdóttir et al., 2015; Pfeiffer and Foster, 2013), it has been hypothesised the hippocampus simulates future paths through the environment at key events during navigation, such as at detours (Spiers and Gilbert, 2015). Consequently, detours requiring simulation of a much larger future route will evoke greater demands on the hippocampus than simulation of shorter routes.

**Figure 1.**
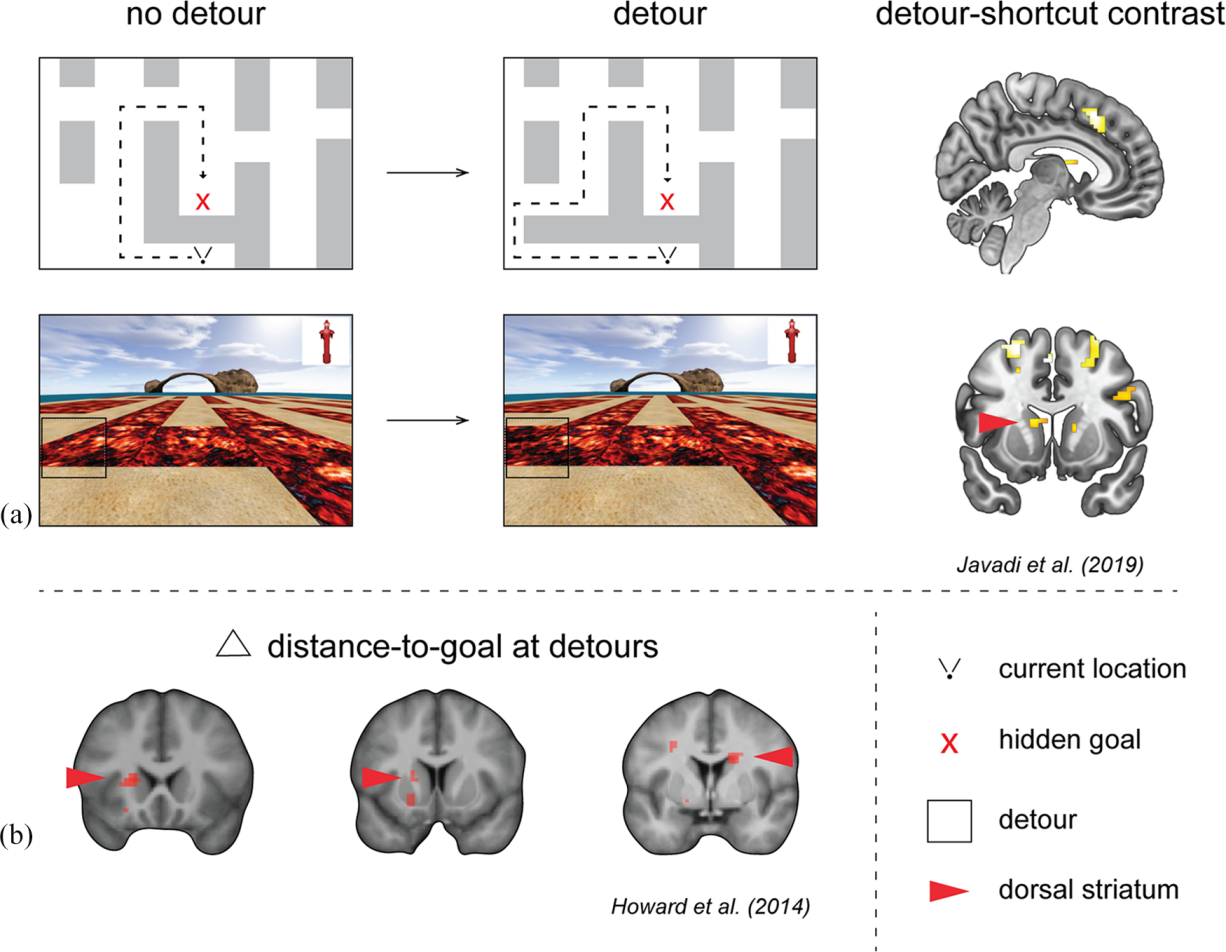
Dorsal striatum activity is correlated with the change in distance to goal at detours. (a) Replotted data from Javadi et al. (2019a) in which fMRI and virtual reality desert island riven with lava was used to examine the brain regions responsive to the change in distance to the goal at detours. Top row shows a zoomed in schematic from the larger virtual environment used and the transition that occurs when the path is unexpectedly blocked. Bottom row: the same change but from the first person perspective. Brain image shows bilateral activity in medial caudate nucleus (dorsal striatum) cluster-corrected for activity correlated positively with the parametrically modulated change in distance-to-goal. (b) Replotted data from Howard et al. (2014). In this study, a film simulation of Soho in London was used to test navigation, including accommodating detours. The amount of change in distance-to-goal caused by forced detours was correlated with the dorsal striatal activity. Red regions show regions activations thresholded at p < 0.005 uncorrected, shown in the mean structural image.

In order to test the prediction of Spiers and Gilbert (2015), a recent study by Javadi et al. (2019a) examined hippocampal response to, respectively, small and large changes in distance to goal at forced detours (see Figure 1(a)). In this task, participants navigated a virtual desert island riven with lava which blocked certain movements across it. Participants first learned the layout and location of several hidden objects, which were later presented as a goal to navigate to. During the test phase, when participants actively navigated the maze, shifts in the location of lava pools either opened up new paths or blocked old paths, resulting in possible shortcuts and detours, respectively. In contrast to the predictions of Spiers and Gilbert (2015), posterior hippocampus did not index the change in distance to goal at detours, but rather prefrontal regions and bilateral caudate nucleus tracked the change in path distance to goal (Javadi et al., 2019). Notably, in Howard et al. (2014), the hippocampal response to distance changes at detours was also accompanied by a similar response in the dorsal striatum (Figure 1(b)). Taken together, these results indicate that the dorsal striatum is more consistent in tracking the change in distance to goal at detours than the hippocampus. This suggests it is timely to reconsider the role of dorsal striatum during flexible navigation and understand how the hippocampus interacts with these regions in cortico-striatal loops (Brown et al., 2012; Goodroe et al., 2018).

## How might the striatum contribute to flexible navigation behaviour?

Despite the traditional role of response learning attributed to striatal function, the striatum has been implicated in studies investigating behavioural flexibility in both rodents and humans, suggesting a more nuanced functionality beyond contributing to a less flexible response system (Johnson et al., 2007). Lesions and inactivations in different areas of striatum produce varied behavioural deficits, indicating a dissociation of respective functional roles (Ragozzino et al., 2002; Sharpe et al., 2019). The striatum is commonly divided up into two anatomically separated regions: the dorsal striatum, composing of the caudate and putamen, and the ventral striatum, composed mainly of the nucleus accumbens although no clear cytoarchitectonic or histochemical boundary between ventral and dorsal striatum exists (Haber and Knutson, 2010). Furthermore, the rodent caudate-putamen is segmented into dorsomedial striatum (homologous to primate caudate) and dorsolateral striatum (homologous to primate putamen) (Cox and Witten, 2019). Early rodent studies did not include strict separation of these regions when using large lesions, which leads to interpretation difficulties (Yin and Knowlton, 2006).

RL models provide a normative framework to investigate neural mechanisms that give rise to flexible and inflexible behaviour (Corrado et al., 2009). Within the RL literature, flexible and goal-directed behaviour is often described by a family of algorithms classified as ‘model-based’. This is commonly contrasted with habitual behaviour described by a separate family of algorithms classified as ‘model-free’ (Dolan and Dayan, 2013; Rusu and Pennartz, 2020). These computational models ‘learn’ states and rewards in the environment by using a component referred to as reward prediction errors, that is, the difference between expected and experienced reward. The goal of a RL agent is to take actions which maximise future reward in the long run (Sutton and Barto, 2018). The canonical finding of reward prediction errors found encoding in single neurons of the ventral tegmental area in the brainstem of macaques (Schultz et al., 1997), a region which has direct dopaminergic projection to the nucleus accumbens in ventral striatum (Haber and Knutson, 2010). Since then, human functional magnetic resonance imaging (fMRI) studies using multi-step decision making tasks have identified ventral striatum as a primary region for the process of reward prediction errors (Daw et al., 2011; Gläscher et al., 2010). Daw et al. (2011) also found the striatal underpinnings of habitual model-free prediction errors and model-based prediction errors overlap in ventral striatum, suggesting the same neural circuitry is involved in both computations. A recent fMRI meta-analysis of multi-step decision making tasks found overlapping regions involved in model-based and model-free computations in globus pallidus and caudate nucleus (Huang et al., 2020).

Beyond the classic divisions of model-free and model-based literature in decision-making tasks, there are other families of RL algorithms that provide alternative accounts, including hierarchical RL, linear RL, and successor representation (Botvinick et al., 2009; Dayan, 1993; Gershman, 2018; Piray and Daw, 2019; Russek et al., 2017; Stachenfeld et al., 2017; Tessereau et al., 2020). In particular, successor representation can account for flexible behaviour of rats and humans in complex mazes (De Cothi et al., 2020) and humans in reward devaluation protocols (Momennejad et al., 2017). Interestingly, components of the successor representation during simulations show similarities to properties of place cells and grid cells, including the influence of goal locations on place field over-representation observed in specific paradigms and influence of environmental geometry on grid field integrity (Duvelle et al., 2019; Ekstrom et al., 2020; Krupic et al., 2015; Stachenfeld et al., 2017). It is an interesting future direction for studies to investigate the relationship between neural responses and the internal computations of successor representation shown to account for behaviour flexibility particularly in some spatial navigation tasks (Russek et al., 2017; for review see Momennejad, 2020). Recent work with rats navigating between four interconnected rooms has revealed that during initial adaptation to pathways being obstructed place cells in CA1 do not adapt their firing fields to accompany the changing behaviour (Duvelle et al., 2020) as might have been predicted by a model in which place cells support SR coding (Stachenfeld et al., 2017). It may be that more stereotyped trajectories would lead to shifts in the place fields as a result of topological manipulations.

The dorsal striatum has commonly been linked to stimulus–response association, or habits, in spatial navigation tasks using human fMRI. Doeller et al. (2008) employed a virtual object-memory task inspired by the Morris water maze. They found activity in caudate nucleus to be parametrically modulated by the influence of intramaze landmarks on goal locations, while right posterior hippocampus correlated with boundary-related influence on goal locations (Doeller et al., 2008). In another study in which participants navigated a virtual town, caudate activity was preferentially active during route following trials, while anterior hippocampus was preferentially active during wayfinding trials (Hartley et al., 2003). Likewise, Iaria et al. (2003) found place strategy use in an eight-arm radial maze task was associated with increased right hippocampal activity while non-spatial response strategy use was associated with increased activity in caudate nucleus. These studies suggest a dissociation between the roles of dorsal striatum and hippocampus for habitual and flexible behaviour, respectively. However, contextual demands may elucidate a more nuanced role for the striatum in multiple behavioural control circuits (Balleine et al., 2015; Ferbinteanu, 2019; Rusu and Pennartz, 2020; Woolley et al., 2015).

In rodents, the involvement of dorsal striatum in both flexible and habitual behaviour could be resolved by considering the functional distinction of dorsolateral and dorsomedial regions (Gasser et al., 2020; Regier et al., 2015; Thorn et al., 2010; Van Der Meer et al., 2010). Studies investigating the homologous regions in humans are made difficult by the lack of spatially precise recordings of neuronal activity. One account suggests dorsal striatum performs the role of an ‘actor’, while ventral striatum performs the parallel role of a ‘critic’ in the ‘actor-critic’ RL framework (Sutton and Barto, 2018). In support of this idea, such a division in computational roles was found during an instrumental learning task using fMRI (O’Doherty et al., 2004). Investigation of functional distinction in dorsal striatum found putamen involvement in habit-based processing from extensive training versus caudate involvement in forward planning (Wunderlich et al., 2012). The role of forward planning at detours could be considered in the task by Javadi et al. (2019a) wherein distance changes were tracked by bilateral caudate nucleus (Figure 1). In a virtual navigation task, Simon and Daw (2011) also found forward planning tracked by striatum using predictions from ‘model-based’ RL.

In a more recent virtual navigation task, Anggraini et al. (2018) identified model-free correlates in dorsal striatum. Model-based correlates were found in the parahippocampus and overlapped with model-free correlates in the retrosplenial cortex. In contrast to Simon and Daw (2011), this study did not utilise visual goal cues and also did not include changes in the maze configuration, more akin to classical spatial navigation paradigms. The different accounts of striatal involvement in prediction errors can perhaps be reconciled by considering that the behavioural strategies and neural mechanisms are not as easily dissociable as previously thought. One spatial planning task found striatal activity related to the difference in path distance between the shortest path and unchosen longest path to goal as a proxy for exhaustive search or forward planning (Kaplan et al., 2017). This indicates that striatal subregions may be involved in planning, which may be the reason these regions are active in different studies. Perhaps a mixed use of strategies is also an underlying reason for this result. Brown et al. (2012) showed that caudate is important for disambiguating context during spatial navigation, together with orbitofrontal cortex and hippocampus. We suggest these findings are in line with a new perspective of these regions. In this view, the caudate encodes learned transition structures. However, the current active transition structure at any point in time is based on the current state of the animal and context within the task, which is proposed to be modulated through cholinergic interneurons in dorsomedial striatum whose task-dependent state information relies on an intact orbitofrontal cortex (Sharpe et al., 2019; Stalnaker et al., 2016). Hippocampus, on the other hand, is involved in learning the structure of the environment (incidental to the task), and also the accompanying association-based learning.

Instrumental learning paradigms in rodents reveal a model-based influence on model-free prediction errors (Langdon et al., 2018). As such, the classical role of dopaminergic prediction errors are more nuanced and can incorporate signals related to behavioural flexibility and the current state of the task in ventral tegmental area (Keiflin et al., 2019; Starkweather et al., 2017) as well as dorsomedial striatum (Stalnaker et al., 2016). Using causal methodology by optogenetically stimulating dopaminergic neurons in ventral tegmental area (the putative cells encoding reward prediction errors), rats could learn associations between cues without endowing them with cached-value, as would be the expected based on pure model-free temporal-difference learning models (Sharpe et al., 2020). Another instrumental learning task found an increasing number of neurons encoding task-relevant information in dorsolateral striatum more so than dorsomedial, suggesting the former may be encoding the development of a habit-based response (Kimchi et al., 2009). Recordings in rats navigating a T-maze found that neurons in dorsomedial striatum were primarily active while choosing between alternative actions after cue-onset, in contrast with neurons in dorsolateral striatum which were primarily active during action execution (Stalnaker et al., 2016; Thorn et al., 2010). found that cholinergic interneurons in rodent dorsomedial—and not dorsolateral striatum—represented information about the current state of the choice task. In addition, this state information was not present in rats with lesions to the orbitofrontal cortex. Taken together, there appears to be shared neural circuitry for model-free and model-based behaviours, and prediction errors may convey more information than the difference between experienced and expected reward (Doll et al., 2012). Perhaps the aforementioned human studies can be reconciled with the notion that caudate can support a mixture of model-free and model-based computations depending on the task and context at hand. Caudate nucleus activity can be expected in response to changes in transition structure if it also encodes model-based information regarding the task environment.

These recent findings pose a new question: What is the human dorsal striatum coding that drives these observed changes in activity during navigation? Rodent work on dorsomedial striatum suggests this region is necessary for execution of flexible goal-directed behaviour (Rusu and Pennartz, 2020). Similarly, dorsomedial lesions have demonstrated similar behavioural deficits to that of hippocampal lesions in terms of deficiencies in goal-directed flexible behaviour (Sharpe et al., 2019). For effective flexible behaviour, Sharpe et al. (2019) suggests hippocampus provides information about the environmental structure, while dorsomedial striatum incorporates information about the transition structure into one’s overall world model. In human navigation, novel forced detours are a classic example of a change in the transition structure. If the caudate updates representations of the transition structure, with greater transitional change resulting in greater demand on caudate activity, then this may explain the results of both Javadi et al. (2019a) and Howard et al. (2014), see Figure 1, where the larger the change in distance at detours the greater the caudate activity evoked. By contrast, hippocampus may be required to construct simulations of journeys through the environments (Bendor and Spiers, 2016). Such simulations may have been much richer in the navigation of London’s Soho (Howard et al., 2014), compared with a desert island (Javadi et al., 2019a), explaining the difference in hippocampal engagement.

Entorhinal cortex may also be involved in representing low-dimensional features of environments by extracting basis sets (or eigenvectors of the successor representation), some of which look visually similar to the iconic hexagonal nature of grid fields (Behrens et al., 2018; Stachenfeld et al., 2017). In RL, a ‘model’ of the environment is defined by P(s’|s, a), equal to the probability of transitioning to a future state (s’) given a specific action (a) in the current state (s) (Sutton and Barto, 2018). Lesions during the Morris water maze have shown the entorhinal cortex to be involved in flexible behaviour, as animals have similar behavioural deficits to those of hippocampal lesions in terms of increased swimming latencies to the hidden platform (Hales et al., 2014). One idea is that the entorhinal cortex supports the ability to form general transition structures of any environment and store information about how distant states or locations are related to each other (Behrens et al., 2018; Constantinescu et al., 2016). However, the unique dorsal striatum contribution may be more closely related to how action-outcome associations are represented and which state is transitioned to as a result of a given motor action (Sharpe et al., 2019).

In conclusion, evidence suggests the dorsomedial striatum/caudate nucleus plays a key role in flexible navigation by representing the transition structure of the environment for guiding future actions (Sharpe et al., 2019) and this may explain observed responses at detours where transition structure changes (Howard et al., 2014; Javadi et al., 2019a). Future research will be useful to observe dorsomedial striatal activity in rodents during dynamic changes to the environment’s transition structure and variations in update demands (e.g. detours that require larger or smaller shifts in the route to the goal). It would also be important to examine the interplay between the striatum, hippocampal/parahippocampal structures, and prefrontal cortex during such updating and representation for the structure of the environment (see Momennejad, 2020). The entorhinal cortex has also been proposed to play a role in coding the transition structure of the layout of the environment or stimulus set (Behrens et al., 2018). Understanding how such a code relates to striatal coding of transition structure would be useful for advancing models of the neural systems supporting flexible navigation behaviour.
